# Co-expression of IL-15/IL-15Ra complex enhances NKG2D-CAR T cell-mediated anti-pancreatic cancer immunity by activating the JAK/STAT5 signaling pathway

**DOI:** 10.3389/fimmu.2025.1498706

**Published:** 2025-06-23

**Authors:** Yiran Chen, Chenxu Jin, Dandan Guo, Xinhui Hui, Yuzhou Ji, Yunhe Huang, Min Xue, Yaoxin Gao, Yaojun Ren, Haizhen Lin, Ying Zhou, Wenzheng Jiang

**Affiliations:** ^1^ Shanghai Key Laboratory of Regulatory Biology, School of Life Sciences, East China Normal University, Shanghai, China; ^2^ College of Life Science, Xinjiang Normal University, Urumqi, China

**Keywords:** NKG2D, CAR T, IL-15, pancreatic carcinoma, JAK/STAT5

## Abstract

The application of CAR T therapy has significantly improved the efficacy of hematological tumors. However, there are still some challenges in the treatment of solid tumors, mainly because the complex immune microenvironment affects the proliferation of T cells, making T cells unable to function well. IL-15 has been reported to be a cytokine that can activate T cells and promote the proliferation and survival of T cells, especially CD8^+^ T cells. The complex formed by the high-affinity binding of IL-15 and IL-15Rα can bind to IL-2/IL-15Rβ/γ heterodimer on the surface of T cells, thereby activating downstream signaling pathways in T cells. In this study, we explored the activity of NKG2D-CAR T expressing IL-15/IL-15Rα complex (IL15C) on pancreatic cancer. The results of *in vitro* experiments showed that CAR T cells expressing IL15C had a stronger killing effect on tumor cells and showed a dose-dependent effect. In addition, the proliferation and anti-apoptosis levels of CAR T cells were enhanced after the co-expression of IL15C. IL15C regulates the function of T cells by activating the JAK/STAT5 signaling pathway of T cells. *In vivo* experiments showed that IL15C-NKG2D-CAR T cells could better inhibit tumor growth than the control group. This study provides a new idea for improving the efficacy of CAR T cells in the treatment of pancreatic cancer.

## 1 Introduction

With the development of cancer immunotherapy, chimeric antigen receptor T cell (CAR T) has made significant progress in the treatment of hematological tumors and has long-term therapeutic effects. Currently, CD19-CAR T cell immunotherapy for the treatment of B cell malignancies has been approved by the FDA (1). However, due to the lack of tumor-specific targets and the inhibitory tumor microenvironment (TME) of solid tumors, CAR T cell therapy for solid tumors still faces many challenges (2, 3). NKG2D belongs to the NKG2 family and is a type 2 transmembrane glycoprotein. It is an activating receptor expressed on the surface of various immune cells, such as NK and T cells. NKG2D has eight ligands, including MHC I chain-related molecules A and B (MICA and MICB) and UL16-binding proteins (ULBP1-6). NKG2DL is usually expressed on the surface of damaged, infected and cancerous cells, but not expressed or low expressed in normal cells and tissues (4, 5). This feature makes NKG2DL a potential target for CAR T treatment of solid tumors. Our previous studies have shown that NKG2D-based CAR T had significant tumor treatment effects both *in vivo* and *in vitro* (6, 7).

Although CAR T cell therapy for solid tumors is technically feasible, the anti-tumor activity of CAR T cells is not satisfactory due to the problems of T cells being easily exhausted and having poor proliferation ability at the tumor site. The tumor is also prone to recurrence. The clinical transformation of CAR T cell therapy still faces great challenges (8, 9).

In order to solve the problem of effective expansion of CAR T *in vivo*, one of the current strategies is to combine CAR T therapy with cytokines. Interleukin-2 (IL-2) is the first cytokine approved for T cell immunotherapy (10). However, the clinical application of IL-2 is still greatly hindered due to its short half-life, easy-to-cause T cell exhaustion, and non-specific toxicity (11). Interleukin 15 (IL-15) and IL-2 belong to the same family, so there are many overlapping functions, such as inducing cell proliferation and activity of T lymphocyte or NK cell (12). IL-15 also promotes the differentiation of CD8^+^ T cells into memory T cells in an antigen-dependent manner (13). Our previous study has shown that the downstream signaling pathway of IL-15 can enhance the anti-tumor activity of NKG2D-CAR T cells and promote the generation of memory T cells (14). Although the toxicity of IL-15 may be lower than that of IL-2, some studies have shown that IL-15 can induce the production of a large number of proinflammatory cytokines and may also be involved in the progression of autoimmune diseases (15, 16).

IL-15 receptor α (IL-15Rα) on the surface of antigen presenting cells (APC) is a high-affinity receptor for IL-15. After IL-15 and IL-15Rα bind to form a complex, the complex binds to the IL-2/IL-15Rβ/γ heterodimer on the surface of T cells through trans-presentation. JAK1/3 was phosphorylated in the cell, thereby activating downstream signaling pathways and enhancing the function of T cells (17, 18). Compared with soluble IL-15, IL-15/IL-15Rα fusion protein has a better effect on activating immune cells, and clinical tests of multiple IL-15/IL-15Rα complexes are actively underway (19, 20). The sushi domain of the structure of IL-15Rα has been shown to be a domain that binds to IL-15 with high affinity (21).

Based on this understanding, we combined IL-15 and the sushi domain of IL-15Rα to construct NKG2D-targeted CAR T cell to express the secretory IL-15/IL-15Rα complex. The data of this study showed that secreted IL-15/IL-15Rα complex could enhance the cytotoxic effect of NKG2D-CAR T cells on pancreatic cancer cell lines and could also promote the proliferation and survival of T cells, especially CD8^+^ T cells. Furthermore, it could promote the differentiation of T cells into memory T cell phenotype. At the same time, IL-15/IL-15Rα complex-secreting NKG2D-CAR T also showed superior anti-tumor activity and survival *in vivo*. This modification of CAR structure could improve the therapeutic effect of CAR T cells on pancreatic cancer both *in vitro* and *in vivo*.

## 2 Materials and methods

### 2.1 Cell lines and culture

Human pancreatic cancer cell lines PANC1, PANC28, and SW1990 cells and the human embryonic kidney cell line HEK293T were obtained from the American Tissue Culture Collection (ATCC, USA) and preserved in our lab. All cell lines were cultured in Modified Eagle Medium (DMEM) (Gibco Laboratories, Grand Island, NY) and supplemented with 10% heat inactive fetal bovine serum (FBS) (Gibco Laboratories) and 1% penicillin-streptomycin (P/S). All cell lines were incubated in a humidified incubator with 5% CO_2_ at 37°C.

### 2.2 Construction of plasmid of NKG2D-CAR and 15C-NKG2D-CAR

To target NKG2DLs, a sequence encoding the extracellular domain of human NKG2D, 4-1BB co-stimulatory, and CD3ζ signaling domain were linked by CD8α hinge and transmembrane region to generate the second-generation CAR construction. Full length of human IL-15 and sushi domain of IL-15Rα DNA sequence was cloned into downstream of NKG2D-CAR region via using ribosomal skipping sequence 2A to construct 15C-NKG2D-CAR. Peptide encoding puromycin-resistance gene was subcloned into the empty lentivirus vector to construct control lentiviral plasmid (Mock).

### 2.3 Lentivirus packaging

On the day before transfection, HEK293T cells were seeded in 10 cm dishes. Packaging plasmid vectors (psPAX2, pMD2.G) and lentiviral plasmids were co-transfected into HEK293T cells when the cell confluence was about 80%-90% at a ratio of 5:3:5 with Polyethylenime PEI, MW 25000 (Polysciences, Warrington, PA, USA). After 6–8 h, the culture medium was replaced with fresh DMEM medium. The supernatant was harvested at 48- and 72-hour time periods, filtered through a 45 μm filter, and concentrated by ultracentrifugation at 15,000 rpm for 2.5 h at 4°C. The virus was resuspended in X-VIVO™ 15 medium (Lonza, Switzerland) and stored at -80°C.

### 2.4 Preparation of CAR T cells

T cells were isolated from peripheral blood mononuclear cells (PBMCs) by using Ficoll-Paque PREMIUM gradient centrifugation method (Cytiva, Logan, UT, USA). CD4 and CD8 MicroBeads (Miltenyi, Bergisch Gladbach, Germany) were used to positively select primary T cells from PBMCs according to the manufacturer’s instruction and activated by Transact (Miltenyi, Bergisch Gladbach, Germany) in X-VIVO containing 1% P/S and 200 IU/mL human recombinant interleukin-2 (IL-2, Seaform Biotech, Beijing, China) for 48 hours. After 48 hours, activated T cells were transduced with respective lentivirus at an MOI of 10. Flow cytometry was used to detect the transduction efficiency.

### 2.5 Reverse transcription PCR

The total RNAs were collected from CAR T cells by using TRIzol reagent (Invitrogen, Shanghai, China) according to the manufacturer’s instruction. 1 μg RNA was further reverse transcribed into cDNA by using Hifair 1st strand cDNA synthesis kit (Yeasen Biotechnology, Shanghai, China) according to the manufacturer’s instruction. PCR reactions were performed in T100™ Thermal Cycler (BIO-RAD) and 2 μL cDNA was used as the template. Reaction cycle conditions were as follows: 94°C for 10 min of predenaturation conditions, 32 cycles at 94°C for 30s, 60°C for 30s, and 72°C for 5s. The primer sequences of the IL15C gene were as follows: forward primer, 5′-GCCCTGCCCCCTCGCGAATTCGAGGGCA-3′; reverse primer, 5′-GAATTCTCATCTAATGCATTTGAGAC-3′. The primer sequences of the β-actin gene were as follows: forward primer, 5′-GTACGCCAACACAGTGCTG-3′; reverse primer, 5′-CGTCATACTCCTGCTTGCTG-3′. All experiments were performed in a triplicate.

### 2.6 Flow cytometry

Fluorochrome-conjugated monoclonal antibodies were used to detect extracellular and intracellular markers by using BD LSR Fortessa. BV421 Anti-human CD3 (Biolegend, 317344), FITC Anti-human CD3 (Biolegend, 317306), APC Anti-human CD4 (BD Biosciences, 555349), FITC Anti-human CD4 (BD Biosciences, 555346), PE Anti-human CD8 (eBioscience™, 12-0088-42), PE/Cy7 Anti-human CD8 (eBioscience™, 25-0087-42), APC Anti-human NKG2D (BD Biosciences, 558071), PE Anti-human MICA/B (Biolegend, 320906), APC Anti-human CD69 (Biolegend, 310910), PE/Cy7 Anti-human CD107a (Biolegend, 328618), PE Anti-human PD-1 (Biolegend, 329906), APC Anti-human TIM3 (Biolegend, 345012), APC Annexin V (Biolegend, 640920), PE/Cy7 Anti-human CD45RA (Biolegend, 304125) and FITC Anti-human CCR7 (Biolegend, 353216) were used for extracellular staining following standard protocols.

For intracellular staining, cells were fixed and permeabilized by using BD Cytofix/Cytoperm™ Kit (BD Biosciences) and stained with intracellular antibodies such as PE/Cy7 Anti-human GzmB (Biolegend, 372214), APC Anti-human IFN-γ (Biolegend, 502512), FITC Anti-human IL-15 (R&D Systems, IC2471F) and PE Anti-human BCL-2 (Biolegend, 658708).

The nuclear membrane was permeabilized using Transcription Factor Buffer Set (BD Biosciences). FITC Anti-human Eomes (eBioscience™, 11-4877-41) and PE Anti-human p-STAT5 (eBioscience™, 12-9010-42) were used for intracellular staining.

### 2.7 Cytotoxicity assay

Firefly Luciferase Reporter Gene Assay Kit (Beyotime Biotechnology, Shanghai, China) was used to detect the lysis of target cells according to the manufacturer’s instructions. Luciferase was transfected into PANC1, PANC28 and SW1990. To detect cytotoxicity, target and effector cells were co-cultured at various effector-to-target (E: T) ratios at 3:1, 6:1, and 9:1 for 18 hours. Tumor cells that were cultured without CAR T cells were defined as control. Reporter gene lysis buffer and firefly luciferase assay reagent were added. The relative light unit was measured at 560 nm using FLUOstar Omega (Thermo Fischer Scientific, USA). The lysis was calculated using the following formula: [(value of control-value of sample)/value of control)] × 100%.

### 2.8 Xenograft mouse model

8-week-old female NOD/SCID/γ-chain^-/-^ (NSG) mice were used in this study. 5×10^6^ PANC1 cells stably transfected luciferase (PANC1-luc) were inoculated subcutaneously (s.c.) in the right flank of all the mice. When the tumor reached up to 100-300 mm^3^, mice were divided randomly into groups of 10 and injected 1×10^7^ CAR T cells per mouse via the tail vein (intravenous; i.v.). The bioluminescent imaging (BLI) was measured weekly using the Xenogen-IVIS Imaging System (Caliper Life Sciences, Hopkinton, MA), and tumor volume was assessed using vernier calipers to monitor the growth of the tumor. All animal studies were done in a pathogen-free environment at the Experimental Animal Center of East China Normal University, Shanghai. All animal procedures were approved by the Institutional Animal Care and Use Committee of East China Normal University and conducted in accordance with relevant guidelines.

### 2.9 Statistical analyses

All statistics were performed using GraphPad Prism 10, and data is expressed as mean ± SD. Data were compared using the one-way ANOVA test, two-way ANOVA test, and Area under curve or t-test. Statistical significance levels are indicated by p-value (*p<0.05, **p<0.01, ***p<0.001, ****p<0.0001), and ns denotes not significant.

## 3 Results

### 3.1 Co-expression of IL15/IL15Rα complex enhanced cytotoxicity, activity, and cytokine secretion of NKG2D-CAR T cells *in vitro*

**Figure 1:**
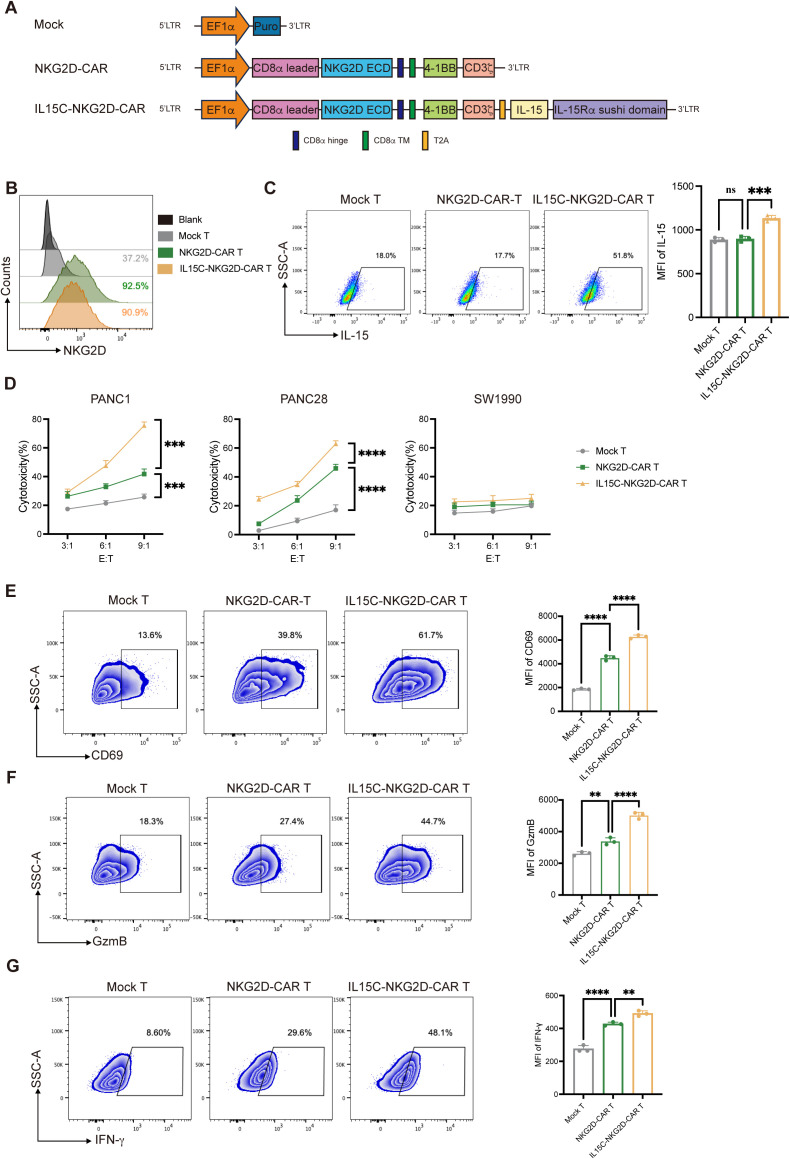
Generation and characterization of IL15C-NKG2D-CAR T cell and cytotoxic activity of CAR T cells. **(A)** Schematic representation of IL15C-NKG2D-CAR T is constructed with NKG2D target, 4-1BB co-stimulatory domain, CD3ζ domain, IL-15 domain and IL-15Ra sushi domain. **(B)** NKG2D-CAR and IL15C-NKG2D-CAR lentiviruses were transduced into primary T cells at an MOI of 10. After 48 hours, flow cytometry was used to measure the transduction efficiency. **(C)** Flow cytometry was used to detect the expression level of IL-15 in T cells by intracellular staining. **(D)** Mock T, NKG2D-CAR T and IL15C-NKG2D-CAR T cell was co-cultured with PANC1 (left), PANC28 (middle) and SW1990 (right) at a different effector to target (E: T) ratios for 18h. The expression of CD69 **(E)**, GzmB **(F)** and IFN-g **(G)** were measured by flow cytometry after co-incubating with PANC1 at a ratio of 6:1 for 18h. MFI were statistically analyzed and shown in column chart (right) (n = 3). **P < 0.01, ***P < 0.001, ****P < 0.0001, ns, not significant.

Previous studies have shown that after IL-15 binds to the IL-15 Receptor α (IL-15Rα), the IL-15/IL-15Rα complex is reversely presented to the IL-2/IL-15 Receptor β/γ (IL-2/IL-15Rβ/γ) on CD8^+^ T cells, which can activate the downstream signaling pathway of T cells and thus enhance the function of T cells (18, 22). Therefore, we designed CAR construction co-expressing IL-15C in order to enhance the effect of T cells against solid tumors. The second-generation CAR backbone which was used to construct CAR T cells contained extracellular construction of human NKG2D (CD134), CD8α hinge domain, CD8α trans-member domain, and the intracellular signaling domains of 4-1BB (CD137) and CD3ζ (**Figure 1A**). The sequence coding fusion protein containing human IL-15 and IL-15Rα sushi domain (IL-15C) was modified on NKG2D-CAR backbone of lentivirus vector through T2A peptide. Mock T cells were generated by transducing lentivirus expressing puromycin resistance gene. CAR T cells were prepared by transducing lentiviruses expressing NKG2D-CAR (NKG2D-CAR T) and IL15C-NKG2D-CAR (IL15C-NKG2D-CAR T).

T cells were isolated from human PBMCs, activated with CD3/CD28 antibodies, infected with lentivirus expressing puromycin resistance gene, NKG2D-CAR, or IL15C-NKG2D-CAR, and the positivity of CAR T cells was detected using flow cytometry by staining of the anti-human NKG2D antibody (**Figure 1B**, **Supplementary Figures 1A-C**). The expression of NKG2D on the surface of CD3^+^, CD4^+^, and CD8^+^ T cells was examined. The results revealed that high levels of NKG2D were present on the surface of CD8^+^ Mock T cells. After transduction with the CAR structure, the mean fluorescence intensity (MFI) of NKG2D on CD8^+^ T cells from the NKG2D-CAR T and IL15C-NKG2D-CAR T groups increased compared to Mock T cells. NKG2D was not detected on the surface of CD4^+^ Mock T cells. However, following infection with the lentivirus, both the expression level and MFI of NKG2D on CD4^+^ T cells in the NKG2D-CAR T and IL15C-NKG2D-CAR T groups increased. These findings demonstrate that both CD4^+^ and CD8^+^ T cells successfully expressed the NKG2D CAR structure (**Supplementary Figure 2**). Mock T, NKG2D-CAR T, and IL15C-NKG2D-CAR T cells were analyzed for the expression of IL-15 after intracellular staining with anti-human IL-15 antibody (**Figure 1C**). The mRNA of CAR T cells was extracted and converted into cDNA, and PCR was used to detect the expression efficiency of IL-15C (**Supplementary Figure 3A**).

The ligand of NKG2D (NKG2DL) on the pancreatic cells’ surface is an important marker related to the killing function of NKG2D-CAR T cells. MICA and MICB are two of the ligands of NKG2D (5, 23). The expression level of MICA/B on several pancreatic cancer cells was detected (**Supplementary Figure 3B**). In order to explore the killing ability of IL15C-CAR T on tumor cells, PANC-1 and PANC-28 which highly express MICA/B were used as target cells, and SW1990 which express low level of MICA/B was used as a negative control. To identify the killing function of CAR T cells, Mock T, NKG2D-CAR T, and IL15C-NKG2D-CAR T were co-incubated with PANC1, PANC28, or SW1990 at different ratios of 3:1, 6:1, and 9:1 for 18 hours separately. The results showed that NKG2D-CAR T had a killing function on pancreatic cancer cells such as PANC-1 and PANC-28 compared with Mock T, and as the effect-to-target ratio increased, the killing effect became more obvious, while there was no significant difference in the killing efficiency of CAR T cells on SW1990 among the three groups (**Figure 1D**). These results indicate that the cytotoxic activity of NKG2D-CAR T against pancreatic cancer cells was significantly enhanced after the co-expression of IL15C on T cells, and the cell lysis of target cells was enhanced with the increase of the effect-to-target ratio.

CD69 is a cell membrane marker that is rapidly expressed after T cells are activated, representing a sign of early activation of T cells. In order to detect the activation effect of IL15C on NKG2D-CAR T, Mock T, NKG2D-CAR T, and IL15C-NKG2D-CAR T cells were co-incubated with PANC-1 at a ratio of 6:1 for 18 hours. Flow cytometry was used to detect the expression level of CD69 on the surface of CAR T cells. The data showed that IL15C-NKG2D-CAR T expressed higher levels of CD69 compared with NKG2D-CAR T and Mock T. Additionally, the mean fluorescence intensity (MFI) of CD69 also had significant differences (**Figure 1E**).

The expression of IFN-γ and granzymes such as granzyme B (GzmB) can increase in the activated T cells, and they play important roles in the cytotoxic function of T cells. To detect the expression of IFN-γ and GzmB, Mock T, NKG2D-CAR T, and IL15C-NKG2D-CAR T was co-incubated with PANC-1 at a ratio of 6:1 for 18 hours, and the expression level of IFN-γ and GzmB in T cells was detected by flow cytometry. The results showed that IL15C-NKG2D-CAR T expressed higher levels of GzmB and IFN-γ than NKG2D-CAR T and Mock T (**Figures 1F, G**). In addition, IL15C-NKG2D-CAR T also expressed higher levels of CD107a, indicating that IL15C increased the degranulation level of NKG2D-CAR T (**Supplementary Figure 3C**). The result suggested that IL15C dramatically enhanced the lytic ability and activation of NKG2D-CAR T cells. These findings revealed that IL15C enhanced the cytotoxicity of NKG2D-CAR T against pancreatic cancer cells by promoting the activation and degranulation of T cells.

### 3.2 Co-expression of IL15/IL15Rα complex promoted the cell proliferation and reduced apoptosis of NKG2D-CAR T cells

**Figure 2:**
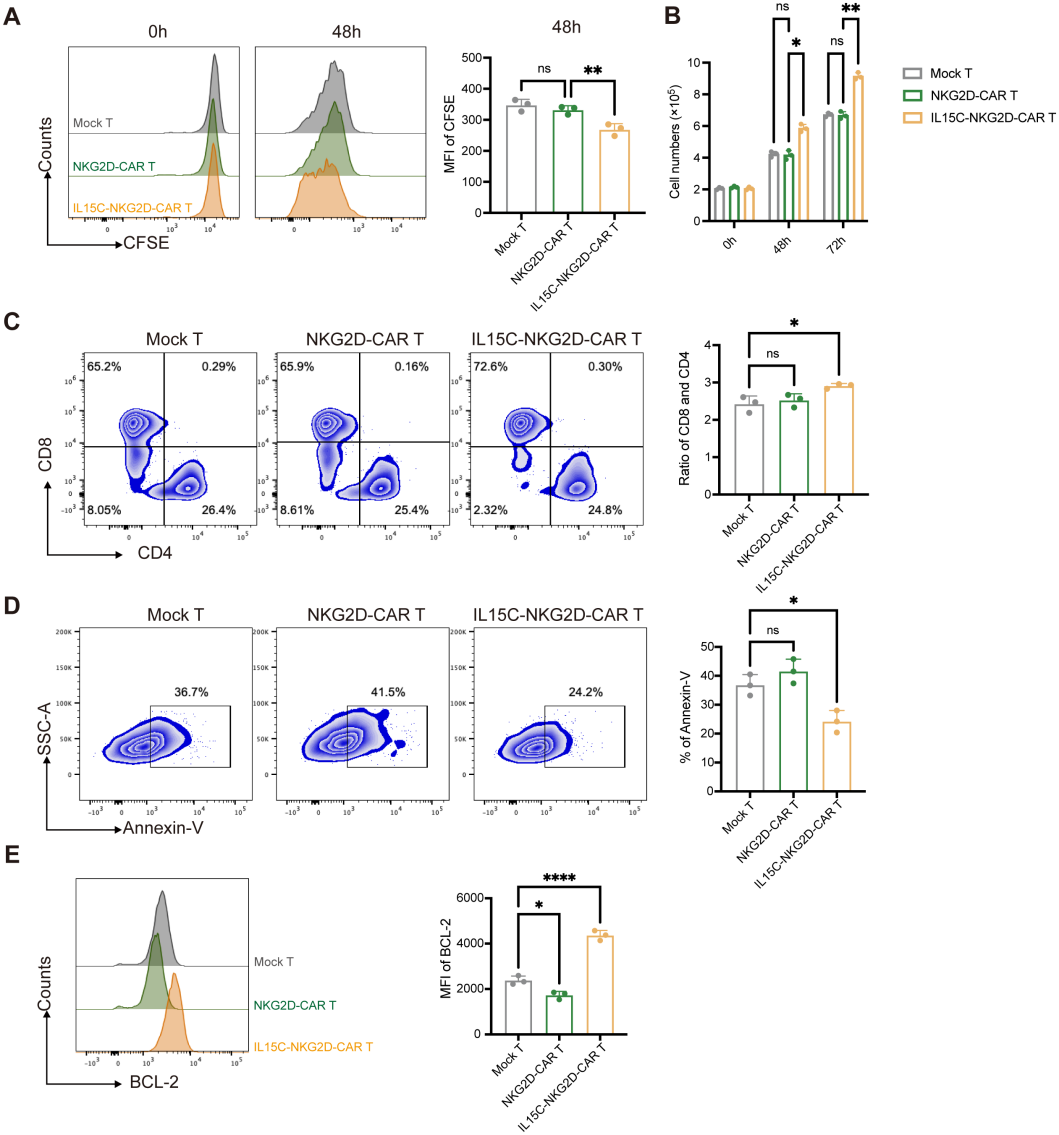
IL5C significantly enhanced the proliferation and anti-apoptosis abilities of CAR T cells. **(A)** Mock T, NKG2D-CAR T and IL15C-NKG2D-CAR T cell were incubated for 72 hours. Proliferative ability of CAR T cell was evaluated by the dilution of CFSE. MFI was statistically analyzed and shown in column chart (right) (n = 3). **(B)** The cell number of Mock T, NKG2D-CAR T and IL5C-NKG2D-CAR T cell was counted at 0h, 48h and 72h. **(C)** The flow cytometric analysis of the ratio of CD8^+^ and CD4^+^ T cells *in vitro* on 72h after being co-incubated with PANC1 at E:T ratio of 6:1. The ratio of CD8^+^ and CD4^+^ T cells was statistically analyzed and shown in column chart (right) (n = 3). **(D)** Mock T, NKG2D-CAR T and IL15C-NKG2D-CAR T cells were cultured without IL-2 for 5 days. Annexin-V was detected by flow cytometry. The percentage of Annexin-V^+^ T cells was statistically analyzed and shown in column chart (right) (n = 3). **(E)** Apoptosis was evaluated by flow cytometry after being co-incubated with PANC1 at E:T ratio of 6:1 for 5 days. The expression level of BCL-2 protein was detected by flow cytometry. MFI was statistically analyzed and shown in column chart (right) (n=3). *P < 0.05, **P < 0.01, ****P < 0.0001, ns, not significant.

IL-15 is one of the T cell growth factors, similar to IL-2, which can promote the proliferation of T cells, especially CD8^+^ T cells (24). In order to detect the expansion of CAR T cells, T cells were labeled with CFSE, and the mean fluorescence intensity of CFSE was detected by flow cytometry at 0 hours and 48 hours. The results showed that the MFI of CFSE was significantly lower in the IL15C-NKG2D-CAR T group than in the NKG2D-CAR T and Mock T group after 48 hours of culture (**Figure 2A**), which indicated that IL15C-NKG2D-CAR T cells proliferated more quickly. In addition, the cell counts were performed at 0 hours, 48 ​​hours, and 72 hours, and similar results were obtained (**Figure 2B**). After CAR T cells were cultured without co-incubation with target cells for 72 hours, the percentage of CD4^+^ and CD8^+^ T cells was analyzed. The results revealed that the CD8/CD4 ratio of IL15C-NKG2D-CAR T cells increased compared with the other two groups, and there was no significant difference between NKG2D-CAR T and Mock T (**Figure 2C**).

After the IL15/IL15Rα complex is presented in trans to IL-2/IL-15Rβ/γ heterodimers on T cells, not only the proliferation of T cells is promoted, but also related signaling pathways are activated, which has an important regulatory effect on the survival and anti-apoptosis of T cells (25). To detect the survival of T cells, Mock T, NKG2D-CAR T, and IL15C-NKG2D-CAR T were cultured for 5 days without IL-2, and apoptosis cells were detected by Annexin-V staining. The results showed that the apoptosis rate of IL15C-NKG2D-CAR T cells was lower than that of NKG2D-CAR T and Mock T cells (**Figure 2D**). These results suggest that IL15C can alleviate the apoptosis of NKG2D-CAR T cells.

To further verify the effect of IL15C on NKG2D-CAR T cell apoptosis, we detected the expression level of BCL-2 in T cells. BCL-2 is a protein located on the mitochondrial membrane, endoplasmic reticulum membrane, and nuclear membrane. BCL-2 can enhance the anti-apoptosis level and survival ability of T cells (26). Previous studies have shown that BCL-2 is a downstream pathway protein of IL-15 (25). The expression level of BCL-2 was detected by flow cytometry after CAR T cells were co-incubated with PANC-1 for 5 days. The results revealed that under the stimulation of target antigen, NKG2D-CAR did not affect the expression of BCL-2 of T cells, but the expression of BCL-2 of IL15C-NKG2D-CAR T was significantly increased (**Figure 2E**). These results illustrated that IL15C could promote the proliferation ability of NKG2D-CAR T cells, especially the expansion ability of CD8^+^ T cells, and enhanced the anti-apoptosis level of T cells and prolonged cell survival as well.

### 3.3 Exhaustion of NKG2D-CAR T cells was reduced and memory cells were formatted by the co-expression of IL15/IL15Rα complex

**Figure 3:**
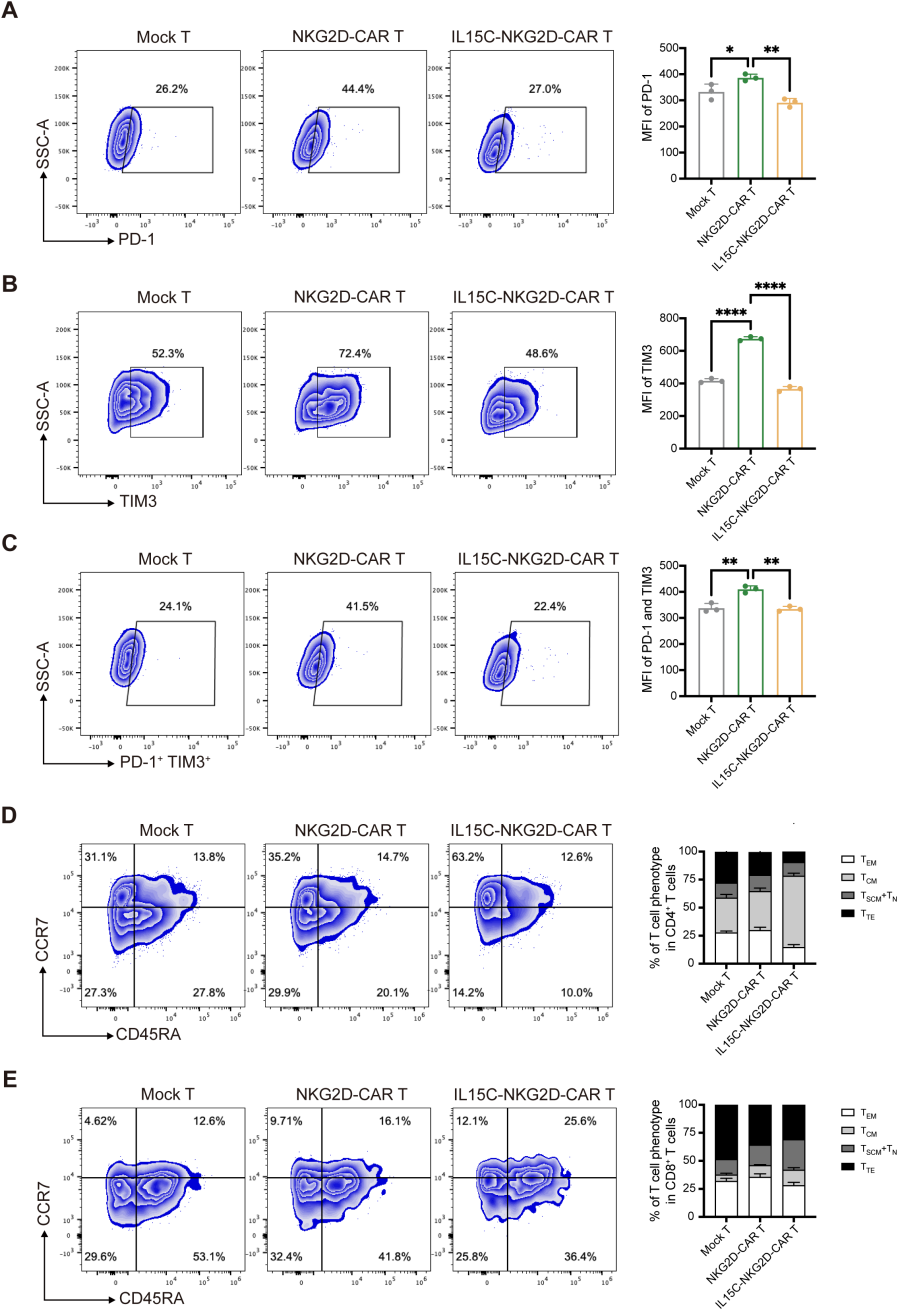
IL15C reduced the exhaustion and influenced the differentiation phenotype. **(A)** Mock T, NKG2D-CAR T and IL15C-NKG2D-CAR T cells were cultured with PANC1 at E:T ratio of 6:1 for 72 hours. Frequencies of PD-1+ T cells, TIM3+ T cells **(B)** and PD-1+ TIM3+ T cells **(C)** was detected by flow cytometry (left). MFI was statistically analyzed and shown in column chart (right) (n = 3). Mock T, NKG2D-CAR T and IL15C-NKG2D-CAR T cells were co-incubated with PANC1 at E:T ratio of 6:1 for 7 days. The percentage of the subpopulation of CD4^+^ T cells **(D)** and CD8^+^ T cells **(E)** was measured by flow cytometry. T_SCM_, CD45RA^+^ CCR7^+^; T_CM_, CD45RA^-^ CCR7^+^; T_EM_, CD45RA^-^ CCR7^-^; T_TE_, CD45RA^+^ CCR7^-^. SCM, memory stem cell; CM, central memory cell; EM, effector memory cell; TE, terminal effector cell. *P < 0.05; **P < 0.01; ****P < 0.0001.

After being stimulated by target antigens, activated T cells express inhibitory receptors such as PD-1 and TIM3, which influence the effector function of T cells and cause T cell exhaustion (27, 28). Previous studies have shown that the signaling pathway downstream of IL-15R can alleviate the exhaustion level of CAR T (14). In order to explore the effect of secreted IL15C on CAR T cell exhaustion, the expression levels of PD-1 and TIM3 were detected by flow cytometry after T cells were co-incubated with target cells for 72 hours. Compared with Mock T, the expression of PD-1 and TIM3 of NKG2D-CAR T cells was significantly increased under the stimulation of target antigens, while the co-expression of IL15C reduced the expression of inhibitory receptors on the surface of T cells, indicating the degree of exhaustion was much lower (**Figures 3A–C**).

In addition, the T cell differentiation phenotype after T cells responded to antigen stimulation was detected and distinguished by CCR7 and CD45RA staining. Central memory T cell (T_CM_) is a less differentiated T cell phenotype that can maintain the long-term survival and effector function of T cells, characterized by CCR7^+^ and CD45RA^-^ (29). The results showed that after antigen stimulation, more IL15C-NKG2D-CAR T cells exhibited a T_CM_ phenotype (**Figures 3D, E**). This observation was also confirmed by the detection of the transcription factor EOMES. IL15C-NKG2D-CAR T expressed higher levels of EOMES compared with Mock T and NKG2D-CAR T (**Supplementary Figure 4**). These results demonstrate that IL15C can promote the differentiation of T cells into memory T cells, thereby promoting long-term anti-tumor activity of CAR T cells.

### 3.4 Co-expression of IL-15/IL15Rα complex regulated the function of NKG2D-CAR T cells via JAK3/STAT5 signal pathway

**Figure 4:**
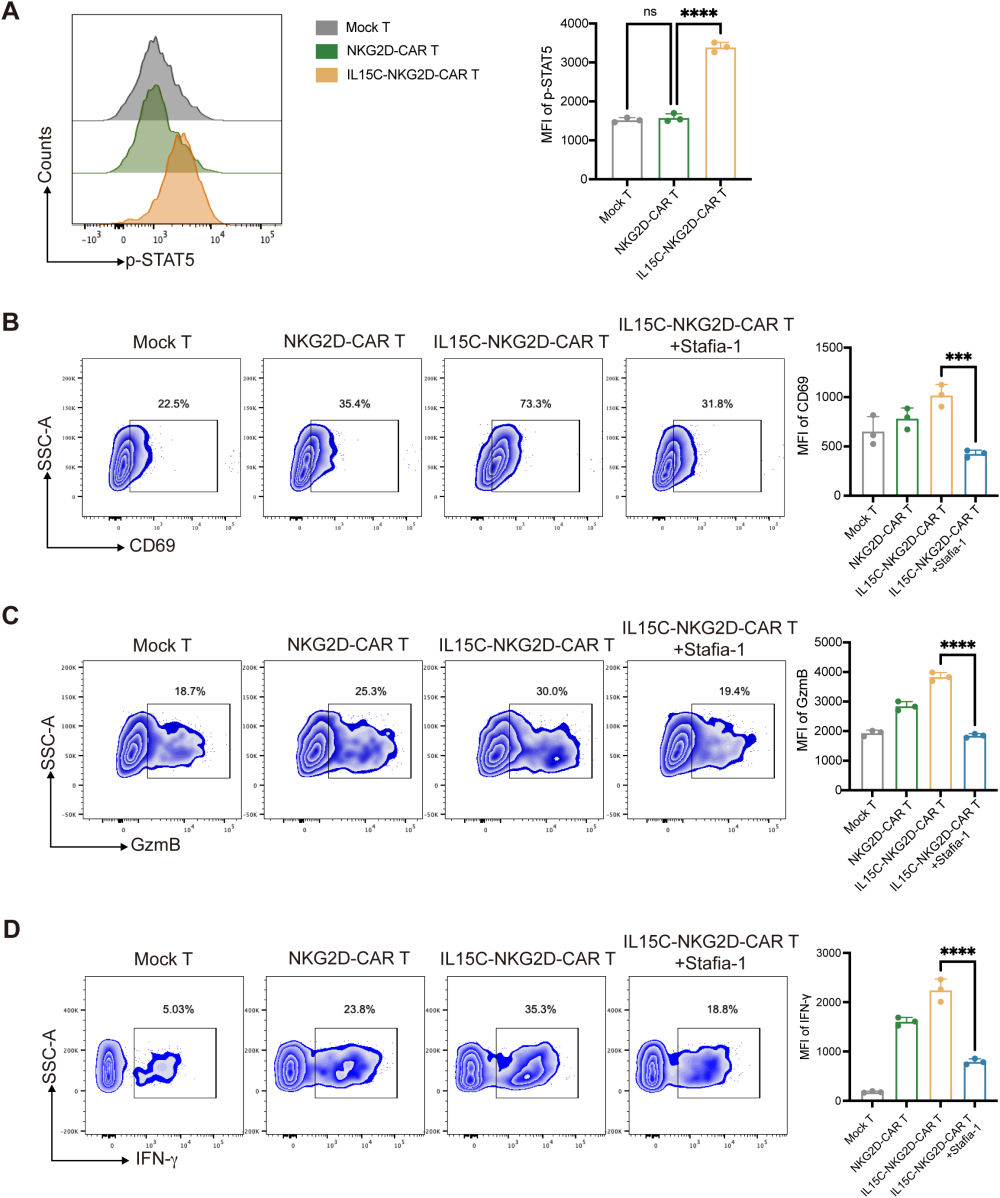
IL15C regulated NKG2D-CAR T cells via JAK3/STAT5 signal pathway. **(A)** Mock T, NKG2D-CAR T and IL15C-NKG2D-CAR T cells were cultured with PANC1 at E:T ratio of 6:1 for 24 hours. The expression level of p-STAT5 was detected by flow cytometry (left). MFI was statistically analyzed and shown in column chart (right) (n = 3). IL15C-NKG2D-CAR T was co-incubated with PANC1 at E:T ratio of 6:1 for 24h with the presence of 50 µm Stafia-1. Flow cytometry was used to detect the expression level of CD69 **(B)**, GzmB **(C)** and IFN-γ **(D)**. MFI was statistically analyzed and shown in column chart (right) (n=3). ***P < 0.001, ****P < 0.0001, ns, not significant.

In order to further explore the mechanism by which IL15C affects T cell function, the signaling pathway downstream of IL-15R was detected. STAT3 and STAT5 are both known transcription factors located downstream of IL-15. Studies have shown that STAT5 activation is more significant after IL-15 stimulation (30). The expression level of phosphorylated STAT5 (p-STAT5) in the nucleus of T cells after antigen stimulation was detected by flow cytometry. The results showed that the expression level of p-STAT5 of IL15C-NKG2D-CAR T was significantly upregulated (**Figure 4A**). In order to verify that IL15C regulates the function of T cells through JAK3/STAT5, the p-STAT5 inhibitor Stafia-1 was added at a concentration of 50 μM (31). The data showed that p-STAT5 was effectively inhibited (**Supplementary Figure 5**). Subsequently, the expression level of the activated markers of CAR T cells after the addition of inhibitor was detected. The results showed that after p-STAT5 was inhibited, the expression levels of CD69, GzmB, and IFN-γ of IL15C-NKG2D-CAR T were significantly downregulated (**Figures 4B–D**). These data suggested that IL15C regulates the function of CAR T cells through the JAK3/STAT5 signaling pathway, thereby enhancing the anti-tumor activity of T cells.

### 3.5 Co-expression of IL15C promoted NKG2D-CAR T-mediated anti-tumor activity in an *in vivo* xenograft model

**Figure 5:**
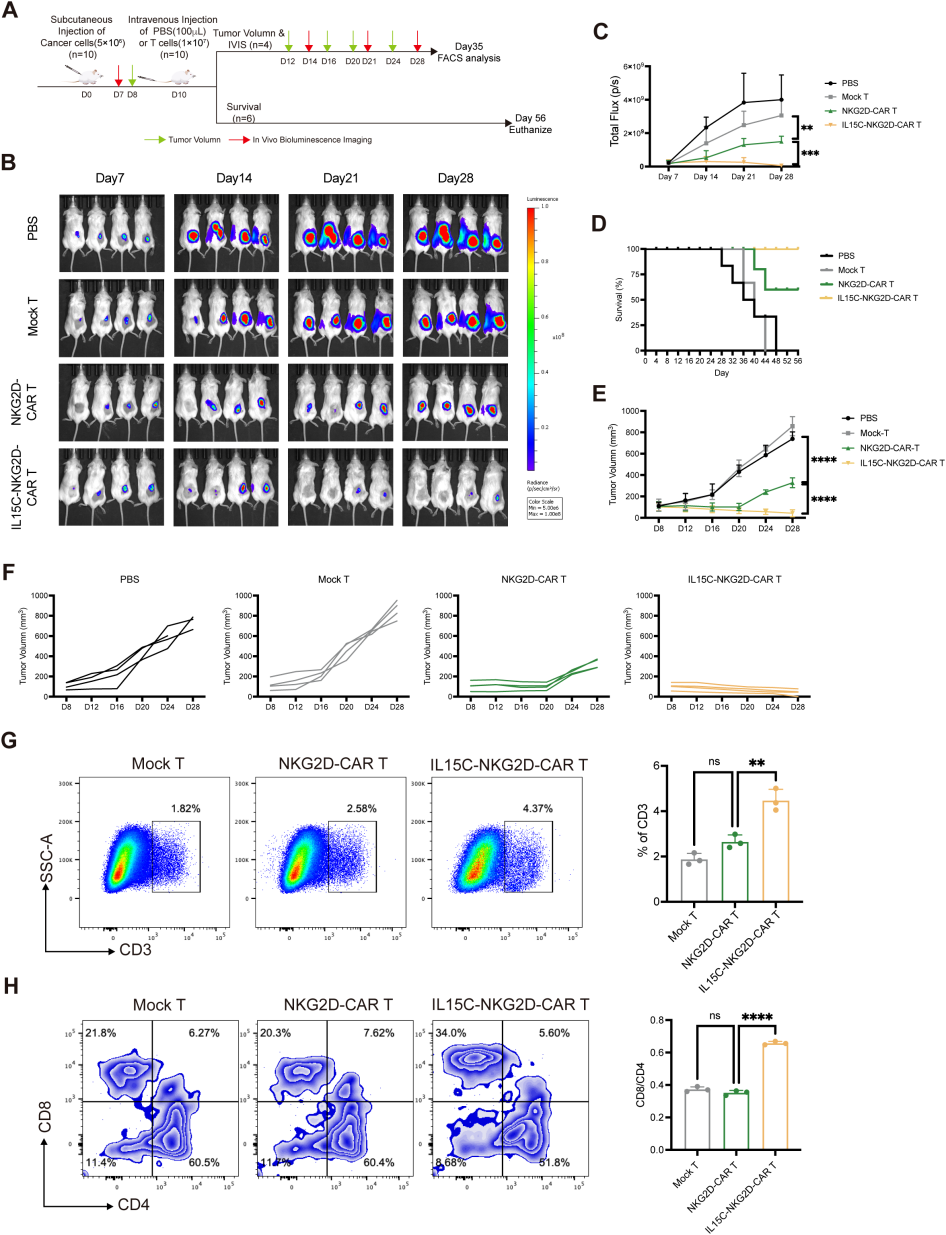
IL15C-NKG2D-CAR T cells inhibited the growth of PANC1 subcutaneous xenografts. **(A)** Schematic outline of *in vivo* experimental design. NSG mice were subcutaneously injected with 5×10^6^ PANC1-luciferase cells. On day 10, mice were randomized and treated with Mock T cells (1×10^7^, n=10), NKG2D-CAR T cells (1×10^7^, n=10), IL15C-NKG2D-CAR T cells (1×10^7^, n=10), or PBS (100μL, n=10) as a control. Mice were divided into two groups on Day 10, one for IVIS imaging and tumor volume measurement (n=4/group), the other for statistical survival curves of mice (n=6/group). **(B, C)** Tumor bioluminescence was monitored using IVIS imaging system at the indicated time points. Data is presented from n=4/group. **(D)** The overall survival data of each group was presented through a Kaplan-Meier survival curve. Data is presented from n=6/group. **(E)** Tumor volume was monitored every 4 days using calipers. **(F)** Each line represents the tumor progression of an individual mouse. **(G)** On day 35, mice were sacrificed and tumor tissues were extracted and prepared as single cell suspension. Cells were stained with anti-human CD3 antibody to determine the percentage of T cells by flow cytometry. **(H)** Anti-human CD4 and CD8 antibody were used to detected the subtype of T cells. The ratio of CD4^+^ and CD8^+^ T cells was calculated and shown in column chart (right) (n=3). **P < 0.01, ***P < 0.001, ****P < 0.0001, ns, not significant.

To evaluate the anti-tumor effect of IL15C-NKG2D-CAR T *in vivo*, subcutaneous xenografts were established by injecting luciferase-transfected PANC1 into NSG mice. On day 10, the mice were randomly divided into groups and injected with PBS, Mock T, NKG2D-CAR T, and IL15C-NKG2D-CAR T, respectively. The results showed that the bioluminescence intensity of the tumors of mice in the PBS group and the Mock T group increased with time. Compared with the PBS group and Mock T group, the NKG2D-CAR T group showed a certain tumor inhibition effect. It is worth noting that the tumors regressed significantly, and the survival period was longer in the IL15C-NKG2D-CAR T group (**Figures 5A–D**). The body weight of mice was measured every four days (**Supplementary Figure 6A**). The results of tumor volume also confirmed a similar conclusion that IL15C-NKG2D-CAR T cells had a more significant control effect on tumors *in vivo* (**Figures 5E, F**, **Supplementary Figure 6B**). On day 35, the mice were sacrificed, the size of the spleen was measured and there was no difference in spleen size between the groups (**Supplementary Figure 6C**). In addition, the proportion of T cells at the tumor site was analyzed and the results revealed that more T cells were detected at the tumor site of IL15C-NKG2D-CAR T group, and more CD8^+^ T cells as well (**Figures 5G, H**). The positive rate of NKG2D-CAR T cells in the tumor site was also tested. CAR T cells at the tumor site still expressed a certain level of NKG2D (**Supplementary Figure 6D**).

### 3.6 Co-expression of IL-15/IL15Rα complex increased the function of NKG2D-CAR T cells at the tumor site

**Figure 6:**
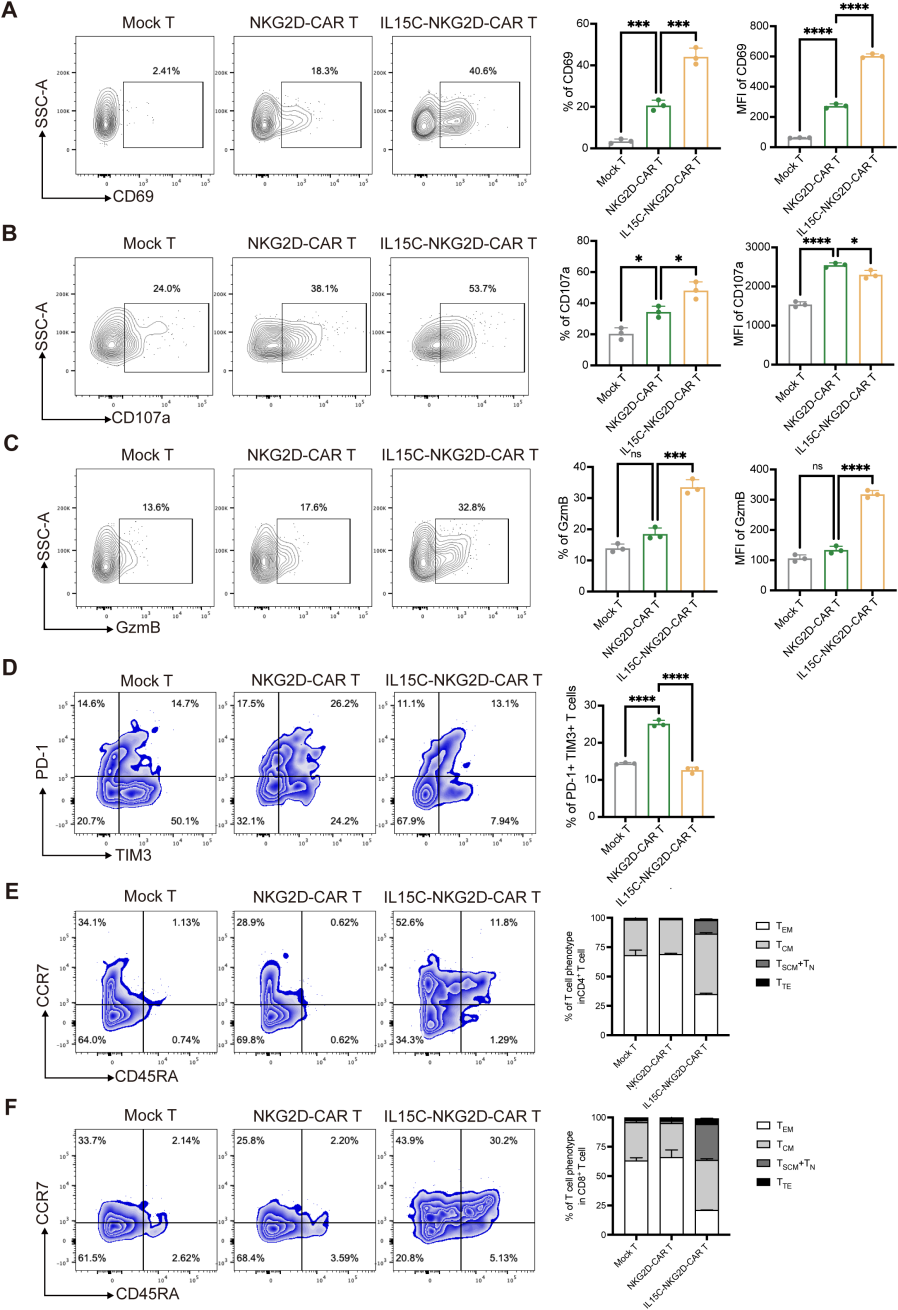
IL15C enhanced CAR T cell function and affected its differentiation *in vivo*. **(A)** On day 35, the tumor tissues were extracted and prepared as single cell suspension. Anti-human CD3 antibody was used to label the CAR T cells. The expression level of CD69, CD107a **(B)**, GzmB **(C)** PD-1 and TIM3 **(D)** was detected by flow cytometry. Percentage and MFI was statistically analyzed and shown in column chart (right) (n=3). The differentiation of CD4^+^ T cells **(E)** and CD8^+^ T cells **(F)** was detected by staining CD45RA and CCR7 antibody. The percentage of T cell subpopulation was shown in column chart (right) (n=3). *P < 0.05, ***P < 0.001, ****P < 0.0001, ns, not significant.

CAR T cells at the tumor site were isolated and analyzed by flow cytometry; the data showed that IL15C-NKG2D-CAR T cells expressed higher levels of cytokines CD69, CD107, and GzmB at the tumor site (**Figures 6A–C**). Inhibitory receptors on the surface of T cells were also detected. The expression level of PD-1 and TIM3 on IL15C-NKG2D-CAR T was significantly lower than that on NKG2D-CAR T at the tumor site (**Figure 6D**). The phenotype of T cells at the tumor site was also analyzed, and the data showed that more memory CD4^+^ and CD8^+^ T cell phenotypes in IL15C-NKG2D-CAR T cells (**Figures 6E, F**). These results suggest that IL15C-NKG2D-CAR T had better anti-tumor activity and persistence at the tumor site.

## 4 Discussion

CAR T cells have made great progress in the field of treating hematological tumors, but the clinical effect of CAR T in treating solid tumors is limited. One of the main reasons is that the complex immune microenvironment of solid tumors leads to poor proliferation and survival of CAR T cells, and CAR T cells are easily exhausted in the body as well. Previous studies have shown that NKG2D-based CAR T cells can produce good tumor treatment effects in immunotherapy (6, 7, 14, 32). Since NKG2D ligands are highly expressed on the surface of most tumor cells but are almost not expressed or expressed at very low levels in normal tissues and cells, CAR T cell therapy targeting NKG2DL has a certain degree of safety (33). In addition, studies have shown that NKG2D ligands are also expressed in tumor blood vessels, immunosuppressive cells (such as Treg and MDSC), and endothelial cells in the tumor microenvironment, which suggests that NKG2D-CAR T can target some cells in the tumor microenvironment while targeting tumor cells, and can relieve the inhibitory effect of the tumor microenvironment to a certain extent (23).

The second-generation CAR structure co-expresses 4-1BB or CD28 co-stimulatory domains in the intracellular segment, and its purpose is to activate CAR T cells in this way. However, due to the complex immune microenvironment of solid tumors, the second-generation CAR T is not so effective in the treatment of solid tumors (2). In order to solve the problem of CAR T proliferation *in vivo*, modifying CAR T cells to secrete cytokines that can activate T cell effects or combining cytokines with CAR T is one of the commonly used methods. IL-2 is the first cytokine used. The combined use of IL-2 and CAR T can overcome the problem of T cell proliferation *in vivo*. IL-2 can not only activate IL-2R downstream-related signaling pathways and enhance T cell activity but also increase the expression of IL-2R on the surface of T cells, further amplifying the activation effect (34). However, IL-2 has a short half-life *in vivo*, which means it has a limited effect on T cell proliferation, and it is prone to T cell exhaustion (35). In addition, studies have shown that IL-2 can promote the function of Treg cells, thereby inhibiting the effect of effector T cells (36). These reasons limit the cell therapy of IL-2 combined with CAR T.

Subsequently, many cytokines were used to enhance CAR T cell therapy, such as IL-7, IL-15, IL-18, IL-21, etc. Previous research of our group has shown that co-expression of IL-7 can enhance the anti-tumor effect of NKG2D-CAR T on prostate cancer (32). IL-15, IL-2, and IL-7 belong to the same family and share the γ chain, so they have a lot of overlap in function (37, 38). Studies have shown that IL-15 can promote the proliferation of CAR T cells and effectively reduce the exhaustion and apoptosis of CAR T cells. The combination of IL-15 and CAR T has a superior anti-tumor effect (39). In addition, IL-15 has been shown to promote the proliferation of CD8^+^ T cells and promote the phenotypic differentiation of T cells into memory types (22, 40). Our previous research also proved that activating the signaling pathway downstream of IL-15R can enhance the anti-tumor effect of NKG2D-CAR T on pancreatic cancer (14).

Although studies have shown that the increase of IL-15 is positively correlated with the survival rate of patients, IL-15 treatment may also cause certain side effects, such as hypotension and thrombocytopenia (12). Consequently, the safety of IL-15 remains to be discussed. IL-15Rα is a high-affinity receptor for IL-15. The fusion protein complex formed by IL-15/IL-15Rα can activate IL-2/IL-15Rβ/γ on the surface of T cells, thereby activating the downstream JAK/STAT signaling pathway and enhancing the proliferation and survival of T cells (21). Studies have shown that the IL-15/IL-15Rα complex has a stronger promoting effect on T cells than IL-15 (41). The strategy of IL-15/IL-15Rα complex to treat tumors has been proven in animal experiments, and clinical research on IL-15 super agonists is also underway (20). In addition, a recent study showed that CD19-CAR T cells secreting IL-15/IL-15Rα complex are less toxic and express lower cytokines (such as IFN-γ), which is related to GvHD, than CD19-CAR T cells that express IL-15; in animal experiments, CD19-CAR T secreting IL-15/IL-15Rα complex can more effectively inhibit tumor growth than CD19-CAR T secreting IL-15; however, liver damage of IL-15/IL-15Rα-CD19-CAR T to mice is greatly reduced, and the score of GvHD is almost 0 (42).

The sushi domain of IL-15Rα is a sequence that binds to IL-15 with high affinity. The affinity of IL-15/IL-15Rα-sushi complex to IL-2/IL-15Rβ/γ is much higher than the affinity of IL-15 to IL-2/IL-15Rβ/γ (20). In this study, we linked IL-15 to IL-15Rα-sushi domain and combined it with the NKG2D-CAR structure to construct an NKG2DL-targeted CAR T cell that can express IL-15/IL-15Rα complex; the expression was detected both in mRNA and protein level and also confirmed by analyzing the downstream JAK3/STAT5 signal pathway. IL-15 can promote the proliferation of T cells, especially CD8^+^ T cells, which was also confirmed in this study (24). The results showed that IL-15/IL-15Rα complex can promote the proliferation of CAR T cells and increase the proportion of CD8^+^ CAR T cells.

IL-15 also affects the regulation of the phenotype of T cell differentiation (43, 44). By detecting the expression of CD45RA and CCR7 of CAR T cells under target antigen stimulation, we proved that the NKG2D-CAR T cells that secrete IL-15/IL-15Rα complex can differentiate more towards the phenotype of memory T cells after antigen stimulation, and similar results were obtained in the *in vivo* experiments. T_CM_ is a group of T cells with long-term memory that can go home to lymph nodes to receive antigen restimulation, which enables patients to achieve immune memory to prevent cancer recurrence (38).

In this study, IL-15 and the sushi domain of IL-15Rα were linked to design a CAR T targeting NKG2DL so that it can recognize NKG2DL^+^ cells and meanwhile express IL-15/IL-15Rα complex. Compared with CAR T cells that directly secrete IL-15, IL15C is safer (42). The results showed that IL15C-NKG2D-CAR T can target pancreatic cancer cells expressing NKG2DL *in vivo* and *in vitro* and exert anti-tumor effects. In addition, the secretory IL-15/IL-15Rα complex can promote the proliferation and survival of CAR T cells, alleviate T cell exhaustion, and promote their differentiation into memory T cell phenotypes, exerting anti-tumor effects for a longer period of time, which has a profound impact on preventing tumor recurrence. In summary, our study provides evidence for the clinical translation of NKG2D-CAR T in the treatment of solid tumors.

This study has several limitations. We selected 4-1BB as the co-stimulatory domain of the CAR structure. CD28 and 4-1BB are commonly used to construct second-generation CAR structures, but due to different antigen stimulation and the complex immune microenvironment, the therapeutic effects of the two co-stimulating molecules are different. To more comprehensively verify the anti-tumor effect of IL15C-NKG2D-CAR T on pancreatic cancer, we will evaluate the stimulatory effects of 4-1BB and CD28 on CAR T cells separately. Other pancreatic cancer cell lines can also be selected for *in vitro* and *in vivo* studies to make the results more comprehensive. In the future, we will select patient samples to construct patient-derived xenografts to verify how IL15C-modified NKG2D-CAR T cells exert anti-tumor effects in PDX models and further promote the clinical translation of our research. Additionally, after mice receive CAR T cell therapy, their tumor growth will be monitored for an extended period to observe whether tumor recurrence occurs, allowing for a better evaluation of the role of IL15C in T cell persistence.

## Data Availability

The original contributions presented in the study are included in the article/**Supplementary Material**. Further inquiries can be directed to the corresponding author.
