# Supplementary material for: Co-expression of IL-15/IL-15Ra complex enhances NKG2D-CAR T cell-mediated anti-pancreatic cancer immunity by activating the JAK/STAT5 signaling pathway
Source: Front Immunol. 2025 Jun 23;16:1498706. doi: 10.3389/fimmu.2025.1498706 (PMC12231003; doi:10.3389/fimmu.2025.1498706)
Supplement: Supplementary file 1 [file DataSheet1.docx]

**A**


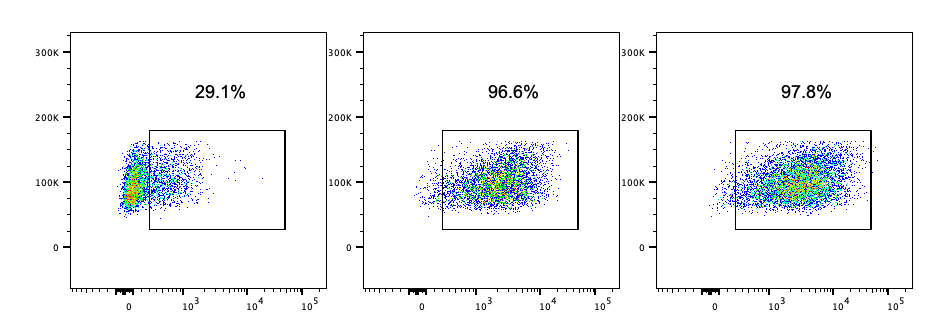

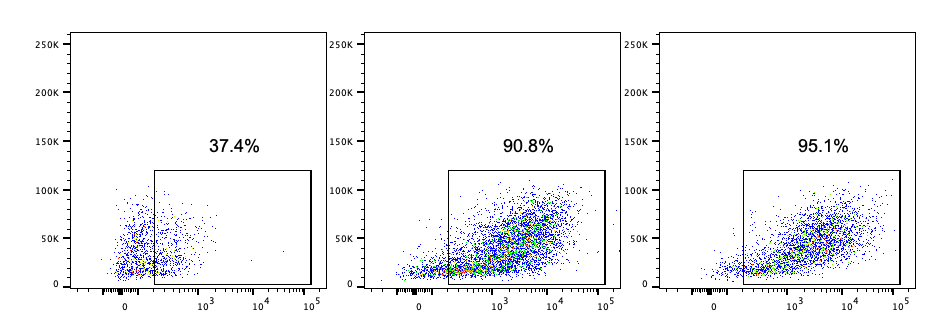

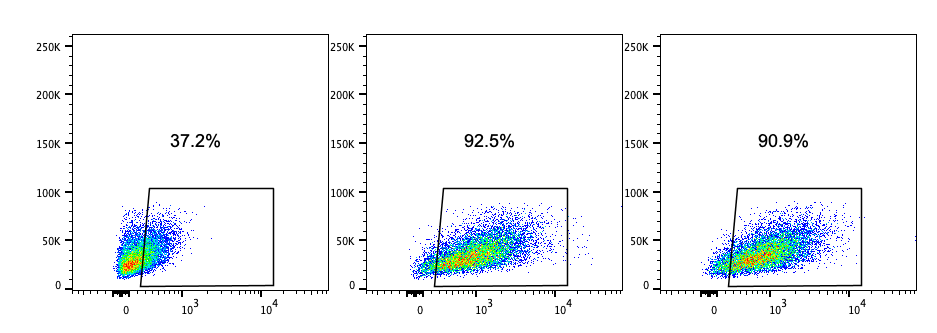

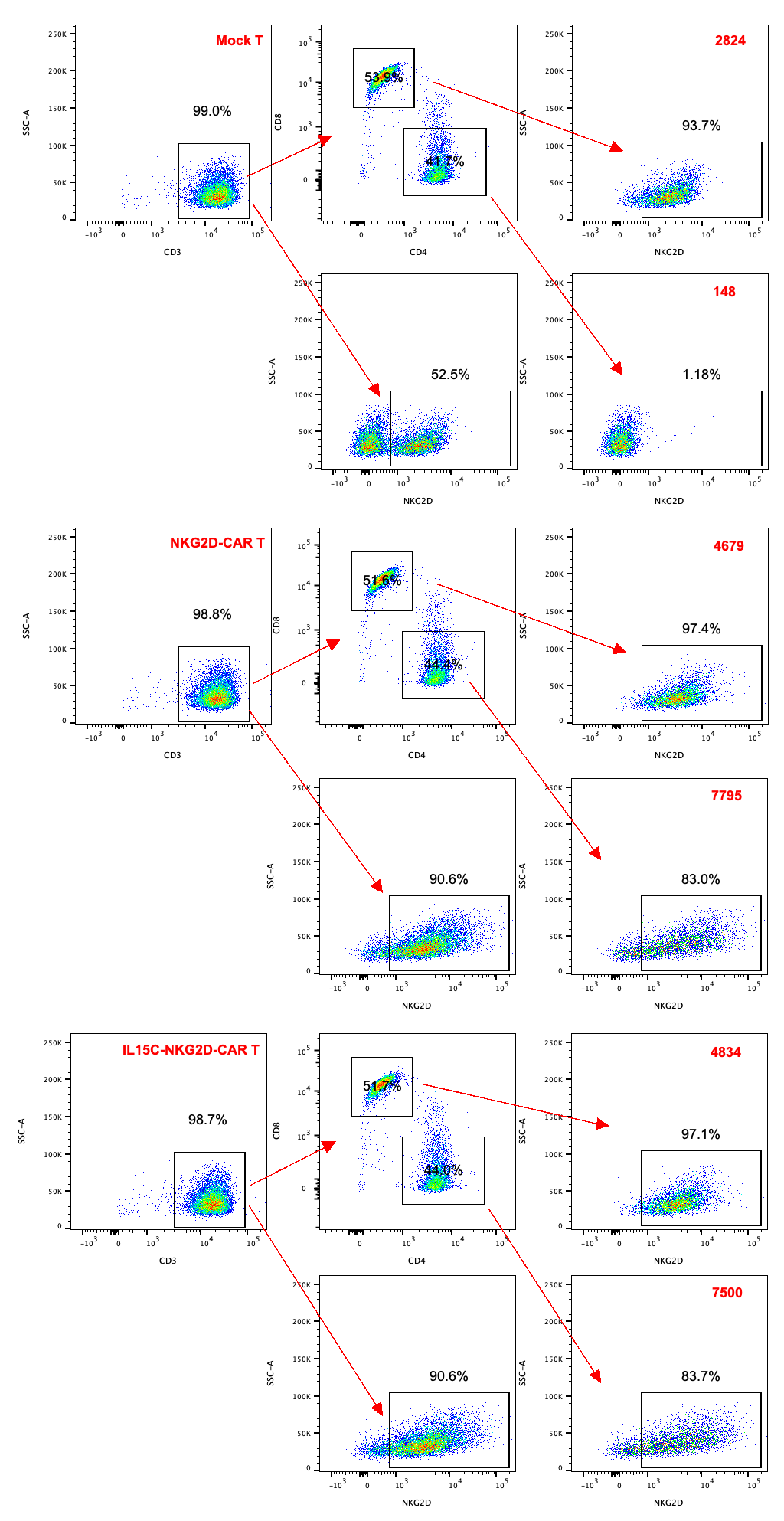


**NKG2D**

**SSC-A**

**Mock T NKG2D-CAR T IL15C-NKG2D-CAR T**

**C**

**Fig. S1 A** Mock, NKG2D-CAR and IL5C-NKG2D-CAR lentiviruses were transduced into primary T cells from healthy donor at an MOI of 10. After 48 hours, flow cytometry was used to measure the transduction efficiency. Experiment was repeated with two other healthy donors (B, C).

**Mock T NKG2D-CAR T IL15C-NKG2D-CAR T**

**NKG2D**

**SSC-A**

**B**

**Mock T NKG2D-CAR T IL15C-NKG2D-CAR T**

**NKG2D**

**SSC-A**

**Fig. S2** The expression level of NKG2D in CD3^+^, CD4^+^, and CD8^+^ T cells was measured through flow cytometry. MFI of NKG2D in CD4^+^, and CD8^+^ T cells was shown separately.


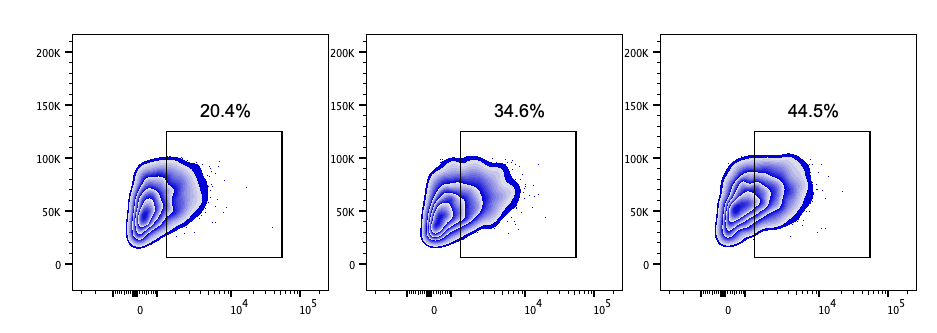

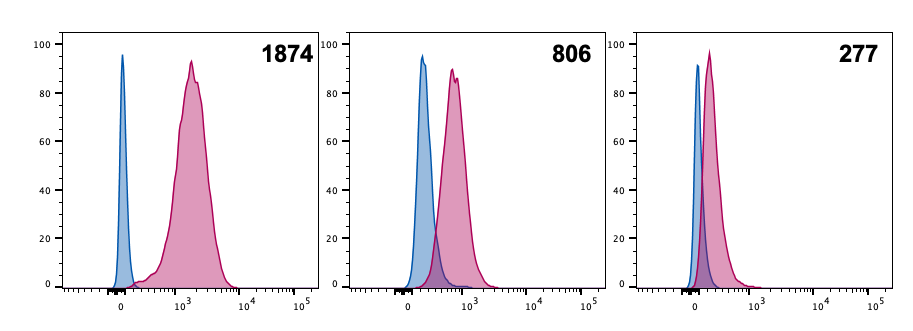


**B**

**A**

**IL15C-NKG2D-**

**CAR T**

**IL15C**

**β-actin**

**Mock T**

**NKG2D-CAR T**


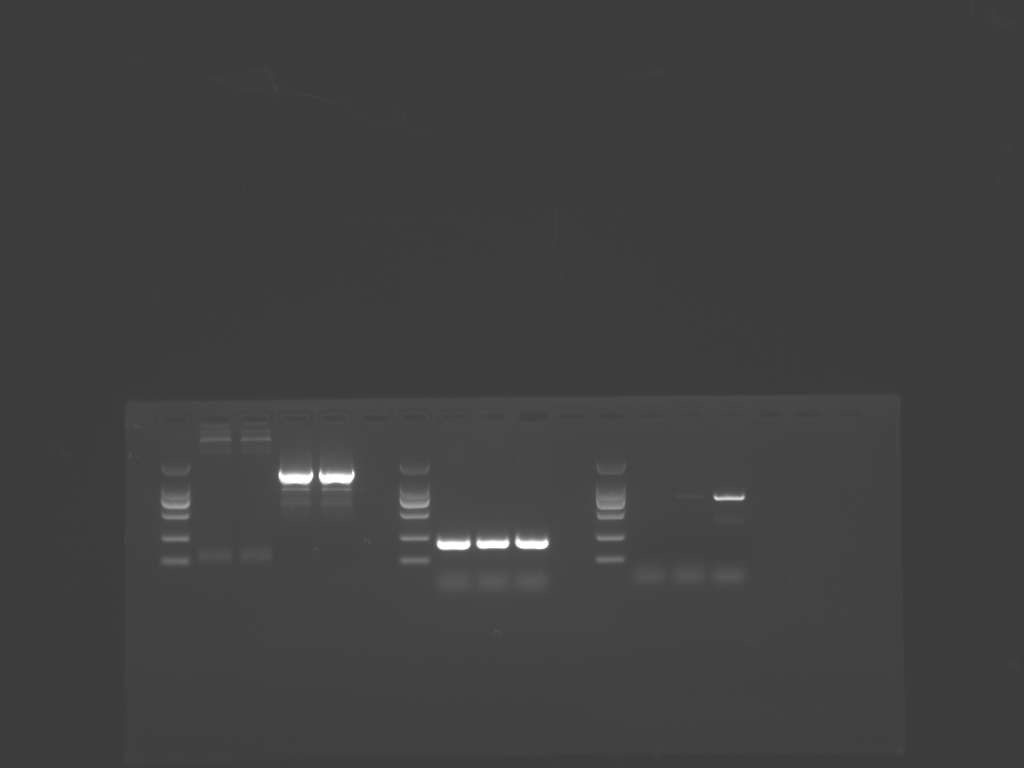

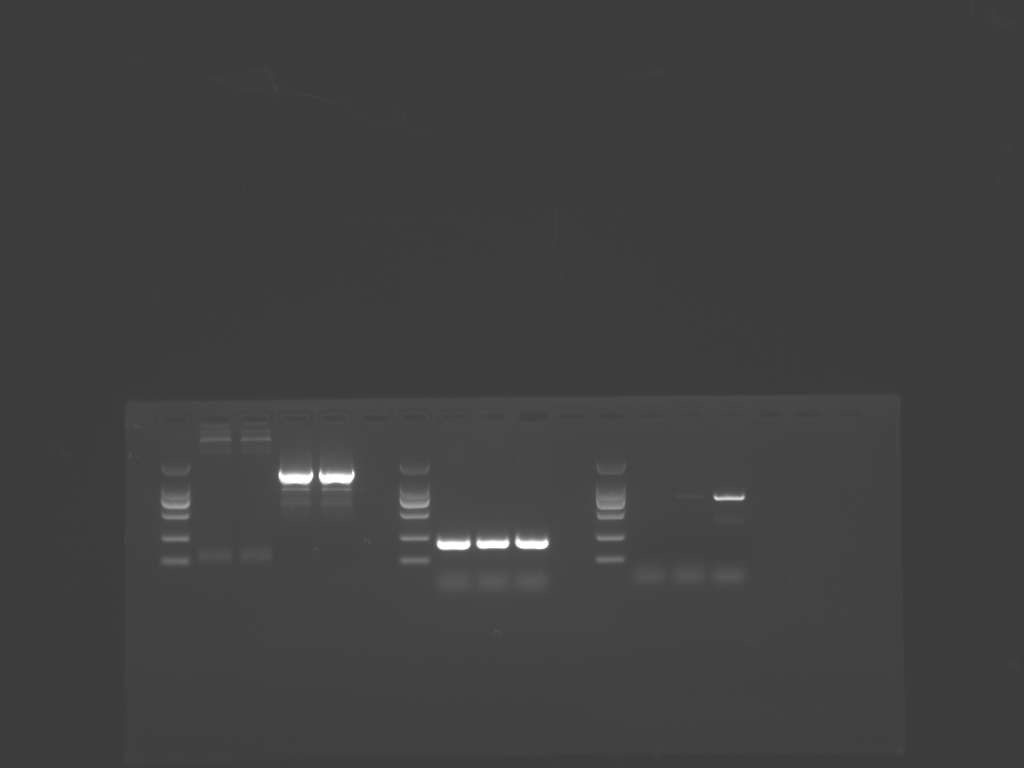


**Fig. S3 A** Mock T, NKG2D-CAR T and IL15C-NKG2D-CAR T cells were collected after being transfected lenti-virus. mRNA of T cells was extracted and converted into cDNA. The expression of IL-15C was statistically detected by PCR, while β-actin was used as control. Relative mRNA expression level of IL-15C was analyzed and shown in column chart (right) (n=3). **B** The expression level of MICA/B on the surface of PANC1, PANC28 and SW1990 was detected by flow cytometry. **C** Mock T, NKG2D-CAR T and IL15C-NKG2D-CAR T were co-incubated with PANC1 at E:T ratio of 6:1 for 18h. Flow cytometry was used to measure the expression of CD107a of T cells. MFI was statistically analyzed and shown in column chart (right) (n = 3). *P < 0.05, **P < 0.01.

**CD107a**

**SSC-A**

**C**

**Mock T NKG2D-CAR T IL15C-NKG2D-CAR T**

**PANC1 PANC28 SW1990**

**MICA/B**

**Counts**


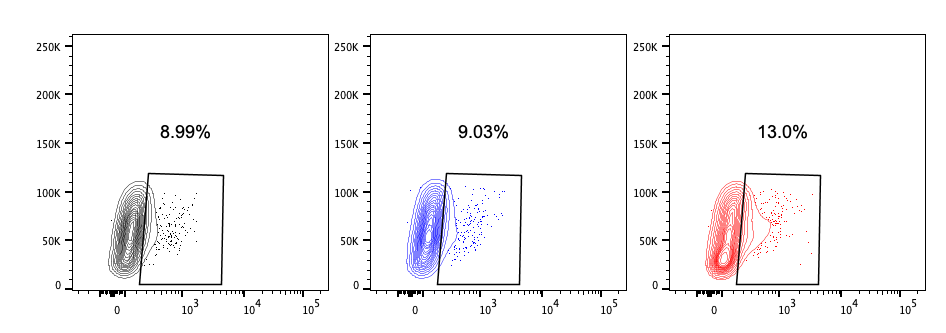


**Fig. S4** Mock T, NKG2D-CAR T and IL15C-NKG2D-CAR T were co-incubated with PANC1 at E:T ratio of 6:1 for 7 days. Flow cytometry was used to measure the expression of EOMES. The percentage of EOMOS^+^ T cell was statistically analyzed and shown in column chart (n = 3). ***P < 0.001, ns, not significant.

**EOMES**

**SSC-A**

**Mock T NKG2D-CAR T IL15C-NKG2D-CAR T**


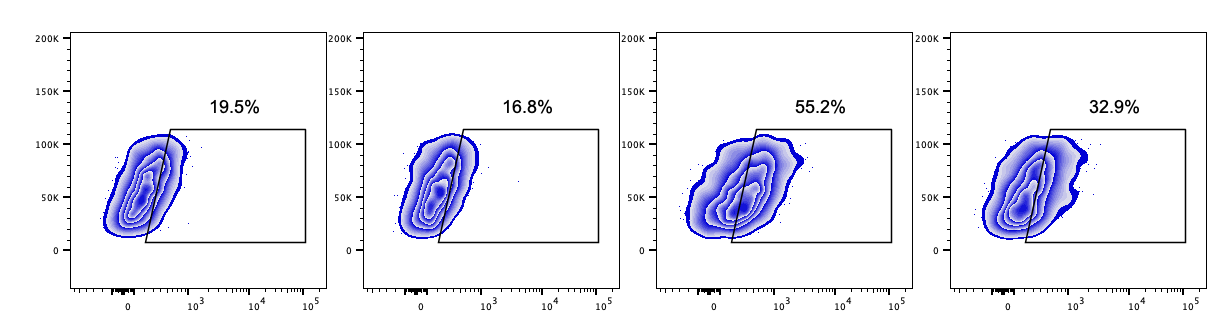


**Fig. S5** Mock T, NKG2D-CAR T and IL15C-NKG2D-CAR T were co-incubated with PANC1 at E:T ratio of 6:1 for 24 hours with or without Stafia-1. Expression of p–STAT5 in the nucleus of T cells was detected by flow cytometry. MFI was statistically analyzed and shown in column chart (n = 3). ***P < 0.001.

**p-STAT5**

**SSC-A**

**IL15C-NKG2D-CAR T**

**+Stafia-1**

**Mock T NKG2D-CAR T IL15C-NKG2D-CAR T**

**A**


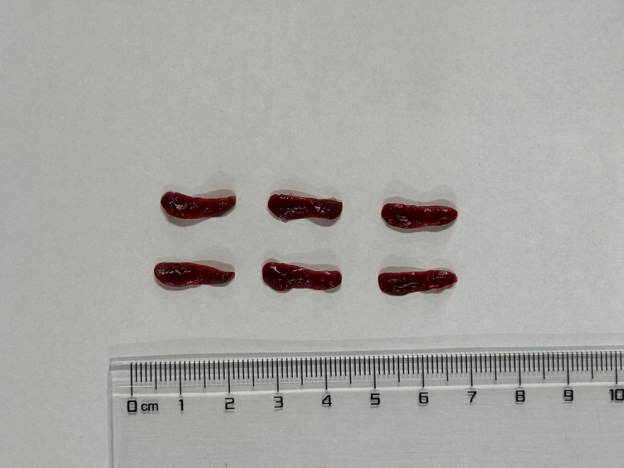

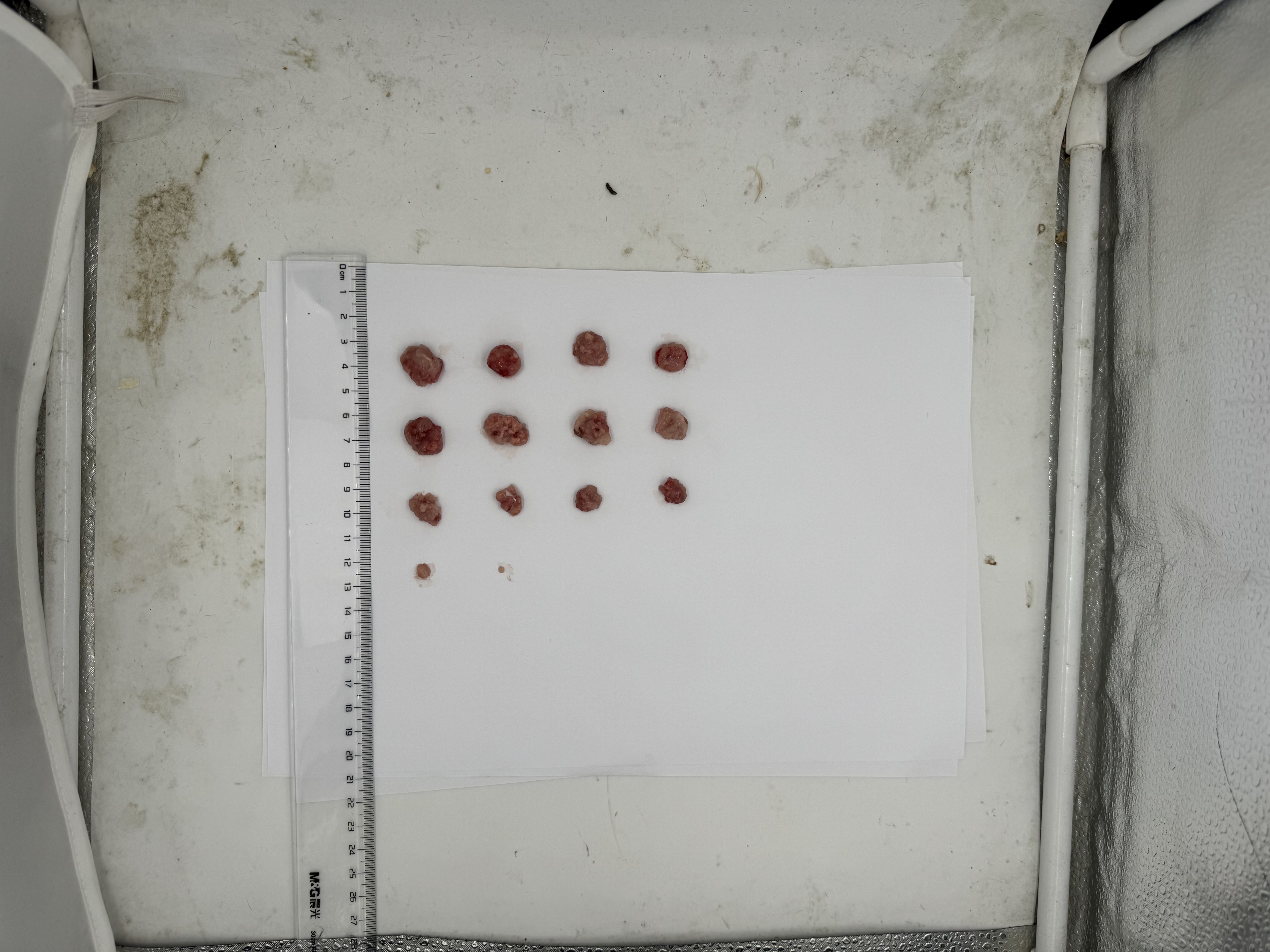

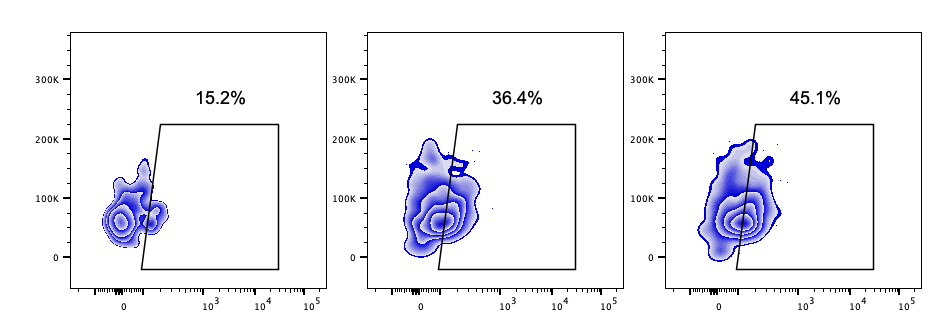


**B**

**D**

**C**

**IL15C-NKG2D-CAR T**

**NKG2D-CAR T**

**Mock T**

**Mock T**

**PBS**

**NKG2D-CAR T**

**IL15C-NKG2D-CAR T**

**NKG2D**

**SSC-A**

**Fig. S6 A** Body weight of mice was monitored every 4 days. **B** On day 35, mice were sacrificed and tumor tissues were extracted. The size of tumor of PBS, Mock T, NKG2D-CAR T and IL15C-NKG2D-CAR T group was measured. **C** The size of spleen of Mock T, NKG2D-CAR T and IL15C-NKG2D-CAR T group was measured as well. **D** Tumor tissues was prepared as single cell suspension. APC anti-human NKG2D antibody was used to detect the expression level of NKG2D of T cells by flow cytometry.

**Mock T NKG2D-CAR T IL15C-NKG2D-CAR T**
